# Supplementary material for: Cortical temporal integration can account for limits of temporal perception: investigations in the binaural system
Source: Commun Biol. 2023 Sep 26;6:981. doi: 10.1038/s42003-023-05361-5 (PMC10522716; doi:10.1038/s42003-023-05361-5)
Supplement: Supplementary file 1 — Supplementary Materials [file 42003_2023_5361_MOESM1_ESM.pdf]

# Supplementary Figures for Cortical Temporal Integration can account for Limits of Temporal Perception: Investigations in the Binaural System

Ravinderjit Singh[1] and *Hari M. Bharadwaj[1,2,3]

[1] *Weldon School of Biomedical Engineering, Purdue University, West Lafayette, IN, United States*

[2] *Department of Speech, Language, and Hearing Sciences, Purdue University, West Lafayette, IN, United States*

[3] *Department of Communication Science and Disorders, University of Pittsburgh, Pittsburgh, PA, United States*

*Correspondence: `hari.bharadwaj@pitt.edu`

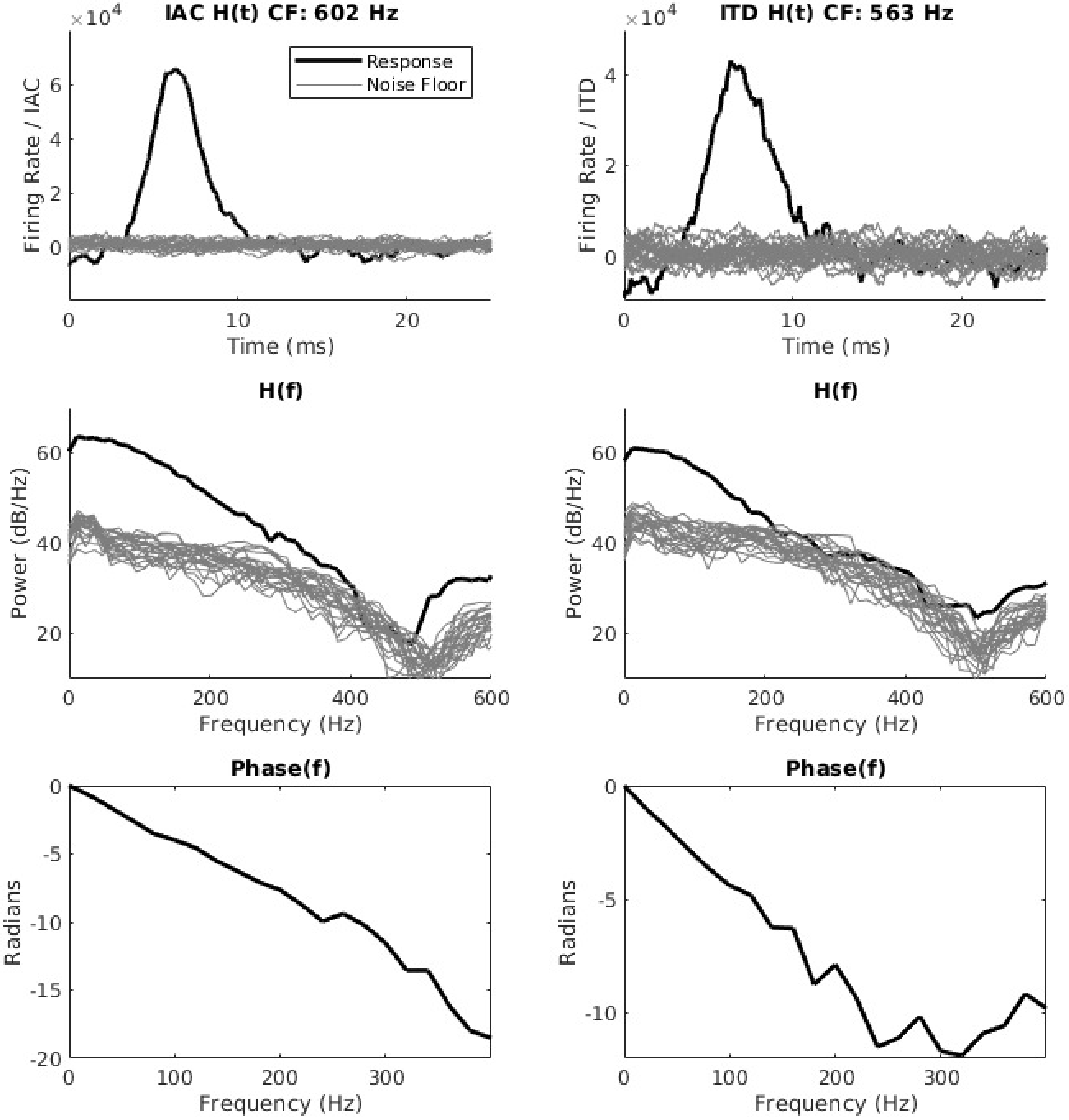


**Supplementary Figure 1. Example system functions for single unit data.** The left column contains an example impulse, frequency, and phase response from a unit with a center frequency (CF) of 602 Hz for IAC and the right column for ITD of a unit with a CF of 563 Hz.

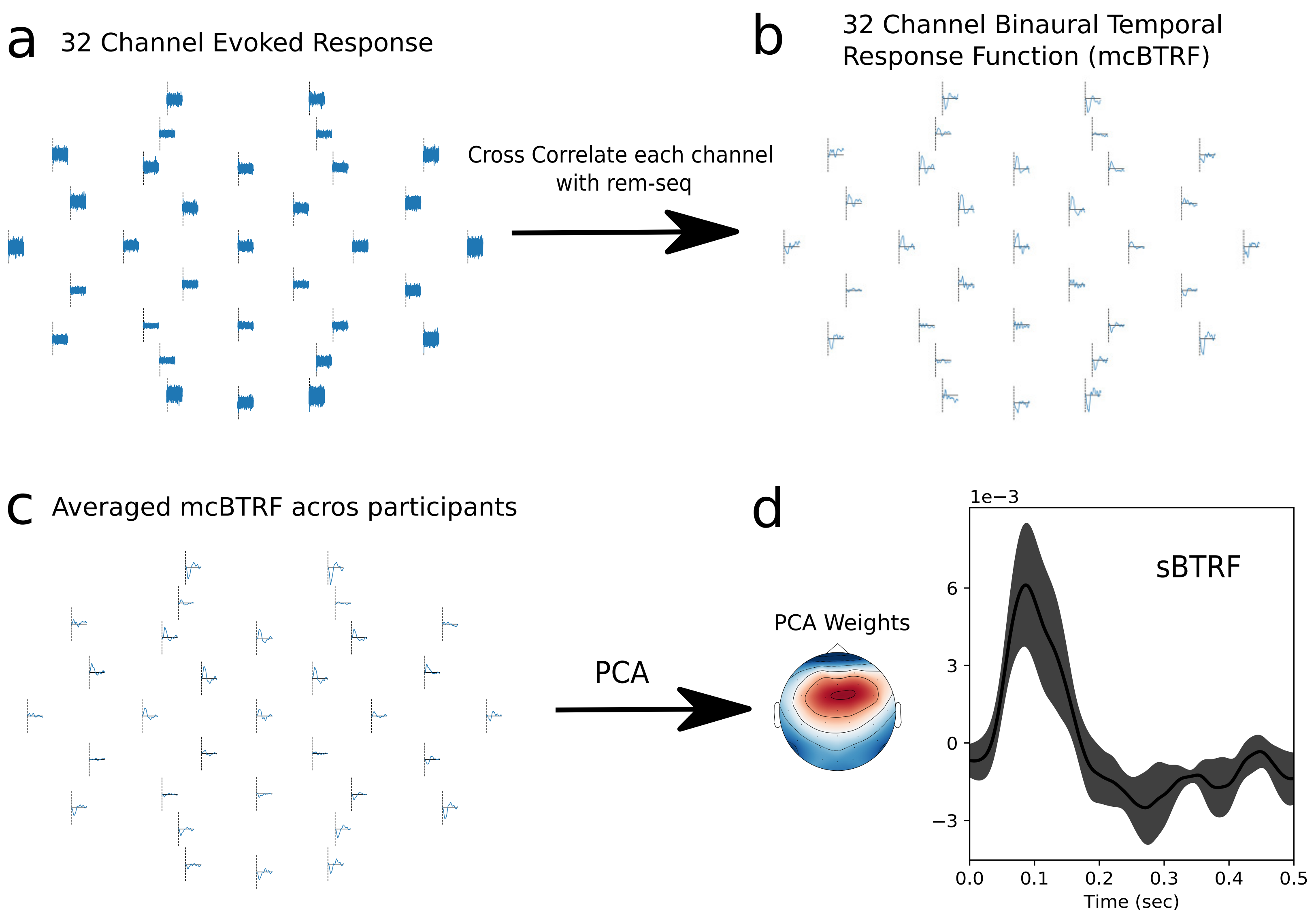


**Supplementary Figure 2. Approach for deriving sBTRF from 32 channel evoked response.** The figure shows the procedure of going from the 32 channel evoked response to the sBTRF. First each channel is cross-correlated with the rem-mseq to obtain the mcBTRF shown in **b**. The mcBTRF was averaged across all participants (**c**), and then PCA was done on averaged mcBTRF. The PCA weights and sBTRF which is the first principal component is depicted in **d**, and the shading is the 95% confidence interval calculated using the standard error computed from jacknifing (n=9 independent samples).

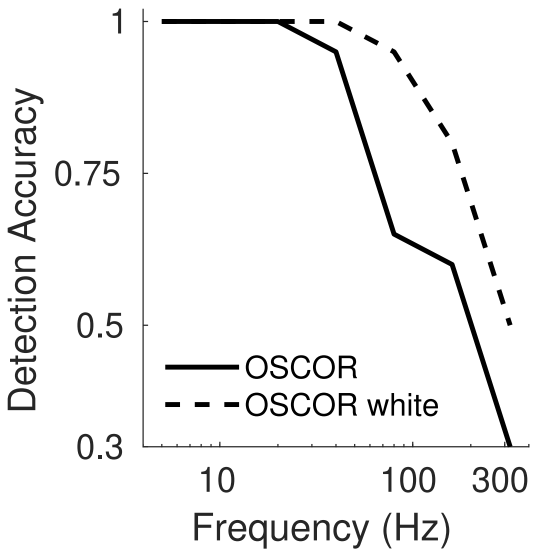


**Supplementary Figure 3. Extension of OSCOR detection with white noise in one participant.** Results from one participant where the OSCOR was measured with the noise band-limited to 0.2-1.5 kHz and with white noise. The OSCOR can be detected out to much higher frequencies with white noise than band-limited noise.

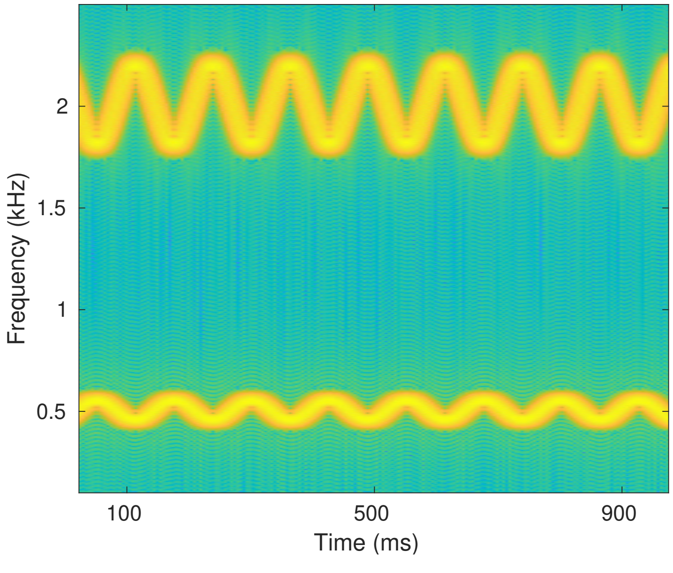


**Supplementary Figure 4. FM phase-difference-detection stimulus.** An example of the FM phase-difference-detection stimulus. In this example, the carriers are at 0.5 and 2 kHz, and the FM rate is 8 Hz with a phase difference of 180 degrees.

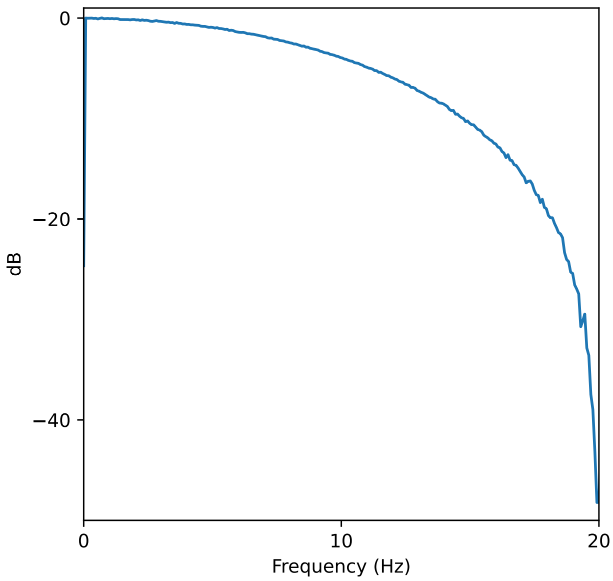


**Supplementary Figure 5. Spectrum of the em-seq.** The spectrum of the em-seq used for EEG data collection in this study.

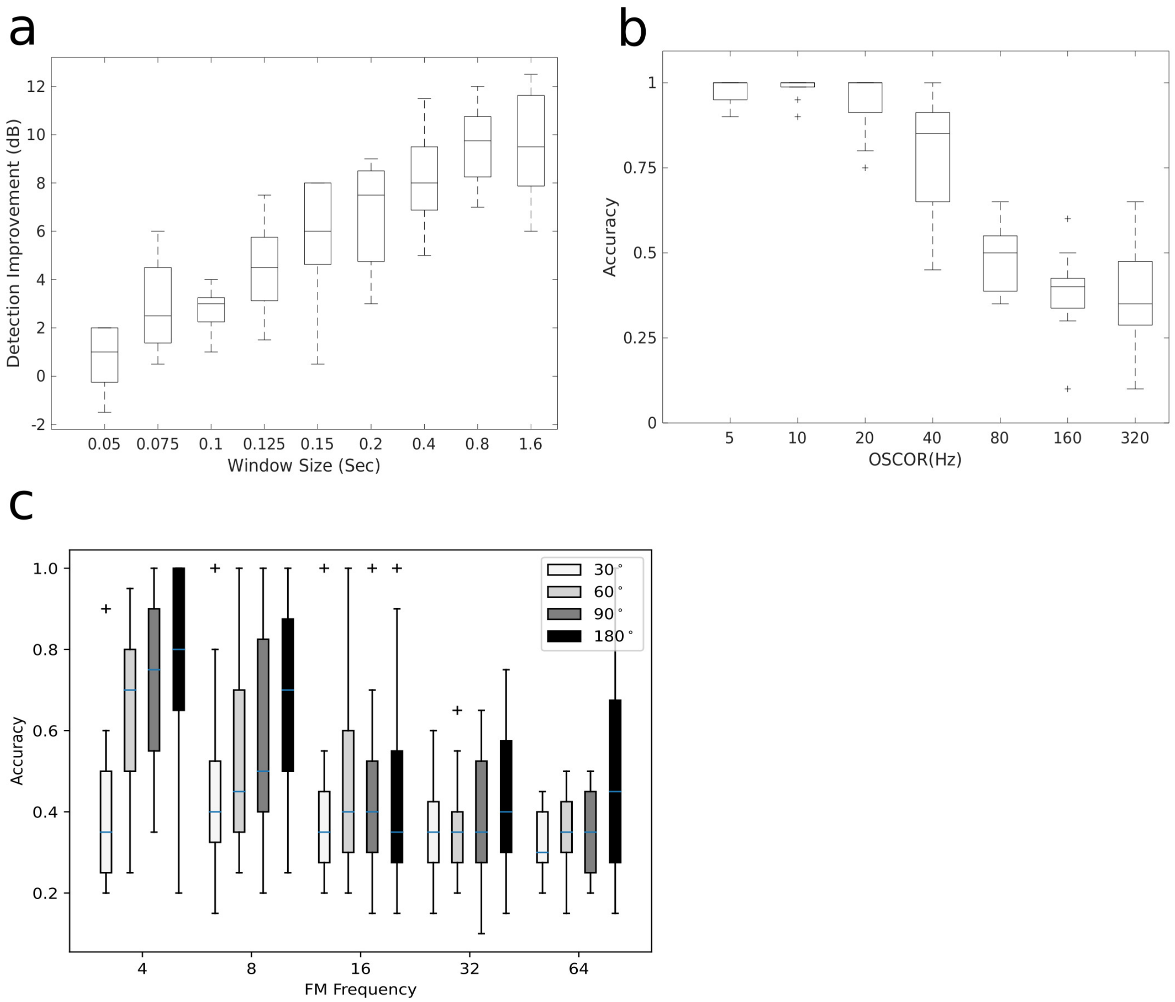


**Supplementary Figure 6. Box plots of behavior data.** Box plots of behavioral data depicted in Figure 4b, 4c, and 4d are depicted in **a, b, c** respectively to help visualize the full distribution of the data. For each box, the line in the middle of the box is the median and the top and bottom of the box are the 75th and 25th percentiles of the sample. The line, i.e. whiskers, that extend out from the box covers the remaining range of the data, except for points greater than 1.5 times the interquartile range from the edge of the box. A '+' is depicted for data greater than 1.5 times the interquartile range.
