# Supplementary material for: Cortical temporal integration can account for limits of temporal perception: investigations in the binaural system
Source: Commun Biol. 2023 Sep 26;6:981. doi: 10.1038/s42003-023-05361-5 (PMC10522716; doi:10.1038/s42003-023-05361-5)
Supplement: Supplementary file 2 — Reporting Summary [file 42003_2023_5361_MOESM2_ESM.pdf]

nature portfolio

Corresponding author(s): Bharadwaj

Last updated by author(s): Sep 10, 2023

# Reporting Summary

Nature Portfolio wishes to improve the reproducibility of the work that we publish. This form provides structure for consistency and transparency in reporting. For further information on Nature Portfolio policies, see our Editorial Policies and the Editorial Policy Checklist.

## Statistics

For all statistical analyses, confirm that the following items are present in the figure legend, table legend, main text, or Methods section.

| n/a | Confirmed | |
|---|---|---|
| ☐ | ☒ | The exact sample size (*n*) for each experimental group/condition, given as a discrete number and unit of measurement |
| ☐ | ☒ | A statement on whether measurements were taken from distinct samples or whether the same sample was measured repeatedly |
| ☐ | ☒ | The statistical test(s) used AND whether they are one- or two-sided<br>*Only common tests should be described solely by name; describe more complex techniques in the Methods section.* |
| ☒ | ☐ | A description of all covariates tested |
| ☐ | ☒ | A description of any assumptions or corrections, such as tests of normality and adjustment for multiple comparisons |
| ☐ | ☒ | A full description of the statistical parameters including central tendency (e.g. means) or other basic estimates (e.g. regression coefficient) AND variation (e.g. standard deviation) or associated estimates of uncertainty (e.g. confidence intervals) |
| ☒ | ☐ | For null hypothesis testing, the test statistic (e.g. *F*, *t*, *r*) with confidence intervals, effect sizes, degrees of freedom and *P* value noted<br>*Give P values as exact values whenever suitable.* |
| ☒ | ☐ | For Bayesian analysis, information on the choice of priors and Markov chain Monte Carlo settings |
| ☒ | ☐ | For hierarchical and complex designs, identification of the appropriate level for tests and full reporting of outcomes |
| ☐ | ☒ | Estimates of effect sizes (e.g. Cohen's *d*, Pearson's *r*), indicating how they were calculated |

*Our web collection on statistics for biologists contains articles on many of the points above.*

## Software and code

Policy information about availability of computer code

| | |
|---|---|
| Data collection | All custom code to generate the stimuli used is available on Github (https://github.com/Ravinderjit-S/DynamicBinauralProcessing) and archived on Zenodo (DOI: 10.5281/zenodo.5780048). There is a very permissive license attached to the code. |
| Data analysis | All custom analysis code is also available on Github (https://github.com/Ravinderjit-S/DynamicBinauralProcessing) and archived on Zenodo (DOI: 10.5281/zenodo.5780048). There is a very permissive license attached to the code. |

For manuscripts utilizing custom algorithms or software that are central to the research but not yet described in published literature, software must be made available to editors and reviewers. We strongly encourage code deposition in a community repository (e.g. GitHub). See the Nature Portfolio guidelines for submitting code & software for further information.

## Data

Policy information about availability of data

All manuscripts must include a data availability statement. This statement should provide the following information, where applicable:

- Accession codes, unique identifiers, or web links for publicly available datasets
- A description of any restrictions on data availability
- For clinical datasets or third party data, please ensure that the statement adheres to our policy

The raw EEG data is openly accessible and archived using Zenodo (DOI: 10.5281/zenodo.5778003).

## Human research participants

Policy information about studies involving human research participants and Sex and Gender in Research.

| | |
|---|---|
| Reporting on sex and gender | Subjects were recruited without reference to sex, gender, race or ethnicity from the greater Lafayette area. The gender split was 7 male and 2 female with the average age being 25 (18-34). No differences in sex, gender, race, or age were used as covariates in any analysis. We also recruited 14 participants from Prolific for an Online portion of the study, but once again no gender, race, or age information was used as a covariate. |
| Population characteristics | Subjects were recruited without reference to sex, gender, race or ethnicity from the greater Lafayette area. The gender split was 7 male and 2 female with the average age being 25 (18-34). No differences in sex, gender, race, or age were used as covariates in any analysis. We also recruited 14 participants from Prolific for an Online portion of the study, but once again no gender, race, or age information was used as a covariate. |
| Recruitment | Subjects recruited through fliers and advertisements in the greater Lafayette area |
| Ethics oversight | IRB and Human Research Protection Program at Purdue University. Protocol Number 1609018209. |

Note that full information on the approval of the study protocol must also be provided in the manuscript.

# Field-specific reporting

Please select the one below that is the best fit for your research. If you are not sure, read the appropriate sections before making your selection.

☒ Life sciences ☐ Behavioural & social sciences ☐ Ecological, evolutionary & environmental sciences

For a reference copy of the document with all sections, see nature.com/documents/nr-reporting-summary-flat.pdf

# Life sciences study design

All studies must disclose on these points even when the disclosure is negative.

| | |
|---|---|
| Sample size | We chose to obtain approximately 9 participants based on existing literature which has studied group averaged EEG responses and found sample sizes between 4 - 12 participants to generally be acceptable to compare group averaged behavioral responses to group averaged EEG responses (as opposed to individual difference comparisons). The key measures undertaken here are completely novel, so a priori estimates of effect size were unavailable. Our results confirm that N=9 participants was sufficient to obtain robust estimates of EEG response and compare to behavioral functions (we note clear separation of our physiological responses from the noise floor after averaging across the 9 participants). |
| Data exclusions | No data were excluded |
| Replication | The key measures in this work are completely novel therefore reproducibility would be best achieved by an independent lab. We provide statistical measures and noise floors to demonstrate that our measures are robust and present with sufficient SNR to clearly separate from noise. Furthermore, the topographic maps for the EEG responses indicate a source consistent with auditory cortex which would be consistent with literature on where the source responding to our stimuli should arise from. We have made all of our data and code openly available to assist with replication and for others to analyze our results. |
| Randomization | All comparisons between physiology and behavior were based on group-level summaries across all participants. There was no splitting into experimental groups calling for randomization. |
| Blinding | Study did not involve interventions. Behavior and physiology were measured from each participant and associations were investigated. |

# Reporting for specific materials, systems and methods

We require information from authors about some types of materials, experimental systems and methods used in many studies. Here, indicate whether each material, system or method listed is relevant to your study. If you are not sure if a list item applies to your research, read the appropriate section before selecting a response.

## Materials & experimental systems

| n/a | Involved in the study | |
|---|---|---|
| ☒ | ☐ | Antibodies |
| ☒ | ☐ | Eukaryotic cell lines |
| ☒ | ☐ | Palaeontology and archaeology |
| ☐ | ☒ | Animals and other organisms |
| ☒ | ☐ | Clinical data |
| ☒ | ☐ | Dual use research of concern |

## Methods

| n/a | Involved in the study | |
|---|---|---|
| ☒ | ☐ | ChIP-seq |
| ☒ | ☐ | Flow cytometry |
| ☒ | ☐ | MRI-based neuroimaging |

# Animals and other research organisms

Policy information about studies involving animals; ARRIVE guidelines recommended for reporting animal research, and Sex and Gender in Research

| | |
|---|---|
| Laboratory animals | Male genetically heterogeneous (i.e., wild-type) chinchillas weighing 400 to 650 grams were used in the study |
| Wild animals | Study did not involve wild animals |
| Reporting on sex | Only male chinchillas were studied here. Our ongoing work includes a balanced sex ratio. |
| Field-collected samples | Study did not involve samples collected in the field |
| Ethics oversight | Animals were used in accordance with protocols approved by the Purdue Animal Care and Use Committee: PACUC Protocol No: 1608001458. |

Note that full information on the approval of the study protocol must also be provided in the manuscript.
